# Evaluating the Resistance of Bifacial Perovskite Photodetectors to Xenon Ion Irradiation

**DOI:** 10.1002/advs.202516418

**Published:** 2025-11-09

**Authors:** Yerassyl Yerlanuly, Hryhorii P. Parkhomenko, Almaz R. Beisenbayev, Maxim V. Zdorovets, Annie Ng, Askhat N. Jumabekov

**Affiliations:** ^1^ Department of Physics School of Sciences and Humanities Nazarbayev University Astana 010000 Kazakhstan; ^2^ Kazakh‐British Technical University Almaty 050000 Kazakhstan; ^3^ Faculty of Physics and Astronomy Adam Mickiewicz University Poznań 61–614 Poland; ^4^ Department of Chemical and Materials Engineering School of Engineering and Digital Sciences Nazarbayev University Astana 010000 Kazakhstan; ^5^ The Institute of Nuclear Physics Almaty 050032 Kazakhstan; ^6^ Department of Electrical and Computer Engineering School of Engineering and Digital Sciences Nazarbayev University Astana 010000 Kazakhstan

**Keywords:** bifacial photodetectors, perovskite, xenon ion irradiation

## Abstract

In this work, high‐performance bifacial perovskite photodetectors (BPPDs) are systematically investigated before and after xenon ion irradiation with energy 1.75 MeV nucleon^−1^ (231 MeV) and fluences up to 10^11^ nucleons cm^−2^, simulating extreme radiation environments. A comprehensive characterization of the materials used in the device's functional layers is conducted, accompanied by an in‐depth analysis of device performance before and after irradiation. Before irradiation, the devices exhibited good performance, with responsivity (*R*) of 0.39 A W^−1^ (front side) and 0.33 A W^−1^ (back side), detectivity (D*) of 2.4 × 10^11^ and 2.0 × 10^11^ Jones. At low fluence (10^10^ nucleons cm^−2^): *R* slightly decreased to 0.37/0.31 A W^−1^, while D* increased to 4.4 × 10^11^/3.7 × 10^11^ Jones due to an increase in shunt resistance. At higher fluence (10^11^ nucleons cm^−2^), irradiation induced high defect formation within the functional layers, causing pronounced degradation: *R* dropped to 0.25/0.18 A W^−1^, D* dropped to 2.8 × 10^11^/2.2 × 10^11^ Jones. Despite these changes, the devices retained over 60% of their initial performance and maintained low spectral noise density, confirming notable radiation tolerance. The synergy of bifaciality and radiation resistance highlights the potential of BPPDs for reliable operation under harsh radiation conditions, offering a promising solution for future optoelectronic applications in radiation‐exposed environments.

## Graphical abstract

Bifacial perovskite photodetectors maintain high efficiency and stability after 231 MeV xenon ion irradiation. Key photodetector parameters remain nearly unchanged at low fluence (10^10^ nucleons cm^−2^) and show only moderate degradation at high fluence (10^11^ nucleons cm^−2^), highlighting their strong radiation tolerance and suitability for optoelectronic applications in extreme radiation environments.

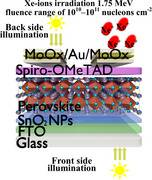

## 1 Introduction

The rapid evolution of optoelectronic technologies has placed increasing demands on materials and device architectures capable of operating reliably in extreme environments.^[^ ^1^, ^2^, ^3^ ^]^ In particular, space‐based and high‐radiation terrestrial applications such as satellite imaging systems, remote sensing platforms, nuclear monitoring equipment, and high‐energy physics experiments necessitate the use of radiation‐tolerant photodetectors with high sensitivity, fast response times, and long‐term operational stability.^[^ ^4^, ^5^, ^6^, ^7^, ^8^ ^]^ Traditional semiconductor materials like silicon and III‐V compounds, while widely used, face limitations in terms of fabrication cost, weight, and degradation under prolonged exposure to ionizing radiation.^[^ ^9^, ^10^, ^11^, ^12^ ^]^ In this context, metal halide perovskites have emerged as a new class of semiconductors that combine excellent optoelectronic properties with low‐cost, solution‐processable fabrication, positioning them as strong candidates for next‐generation photodetectors.^[^ ^7^, ^13^, ^14^, ^15^ ^]^

Among the various perovskite device architectures, bifacial perovskite photodetectors (BPPDs) represent a particularly innovative design.^[^ ^16^, ^17^, ^18^ ^]^ Unlike conventional single‐sided devices, bifacial photodetectors are capable of harvesting light from both front and rear surfaces, enabling higher quantum efficiencies and improved adaptability in diverse lighting conditions.^[^ ^19^, ^20^ ^]^ This dual‐sided architecture is not only advantageous for maximizing photodetection in diffuse or reflective environments but also aligns with the growing trend toward multifunctional and compact device designs. Despite the promising potential of BPPDs, their stability and resilience under radiation exposure remain largely unexplored, presenting a significant gap in the current body of research.

Radiation‐induced degradation presents a formidable challenge for any optoelectronic device intended for high‐radiation environments.^[^ ^21^, ^22^, ^23^, ^24^, ^25^, ^26^ ^]^ Ion irradiation, in particular, poses a severe threat due to its ability to deposit large amounts of energy along localized tracks, causing displacement damage, ionization effects, and defect formation in the material.^[^ ^27^, ^28^ ^]^ These effects can lead to a significant deterioration in device performance, including increased dark current, reduced photoresponsivity, and long‐term functional failure.^[^ ^29^, ^30^ ^]^ While a lot of studies have investigated the radiation hardness of perovskite solar cells and photodetectors,^[^ ^7^, ^11^, ^13^, ^14^, ^29^, ^30^ ^]^ the impact of ion exposure on BPPDs has not yet been systematically evaluated.

In this study, the effect of xenon ion irradiation with an energy of 231 MeV and a fluence range of 10^10^–10^11^ nucleons cm^−2^ on high‐performance BPPDs is investigated. The study provides the ***first systematic investigation of BPPDs under heavy ion exposure***, combining a comprehensive analysis of the materials used in the functional layers with a detailed evaluation of device characteristics before and after irradiation, linking high‐fluence exposure to defect‐induced degradation in both the perovskite absorber and transport layers. The findings not only highlight the notable radiation tolerance of BPPDs but also provide important guidance for the development of next‐generation, radiation‐hardened perovskite optoelectronic devices.

## 2 Results and Discussion

**Figure 1:**
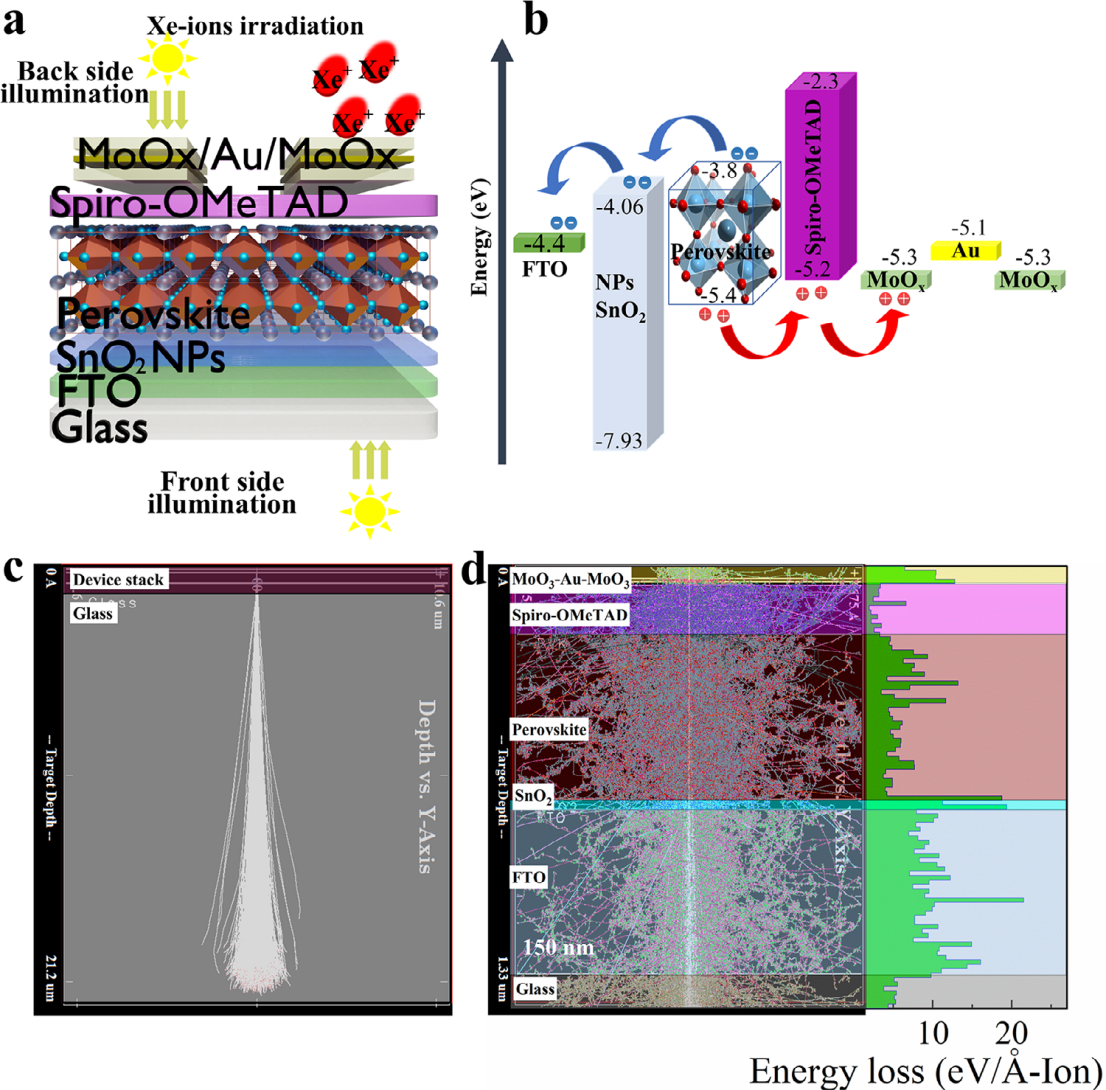
a) Schematic illustration of the BPPD device structure and b) corresponding energy levels diagram. SRIM simulations of 231 MeV xenon ion interactions with the device structure: c) full penetration depth and d) magnified view of the device functional layers.

The schematic structure of the BPPDs with the architecture glass (2.2 mm)/FTO (≈500 nm)/SnO_2_ (≈30 nm)/Cs_0.05_MA_0.23_FA_0.72_Pb(I_0.77_Br_0.23_)_3_ (≈500 nm)/Spiro‐OMeTAD (≈150 nm)/ MoO_3_/Au/MoO_3_ (10/10/35 nm) is shown in **Figure** **1**a. The energy diagram is depicted in Figure 1b. The fabricated devices were irradiated with 231 MeV xenon ions at a fluence range of 10^10^–10^11^ nucleons cm^−2^. A Stopping and Range of Ions in Matter (SRIM) simulation was performed to evaluate the penetration depth and damage profile of the BPPDs. The 231 MeV xenon ion beam was configured for vertical irradiation from the side of the transparent back electrode of the MoO_3_/Au/MoO_3_, similar to the experimental conditions. For the simulation, the measured thicknesses of all functional layers of the device were used, and the material density values were taken from literature sources^[^ ^31^, ^32^ ^]^ and the built‐in SRIM libraries. A first SRIM simulation was carried out to estimate the maximum penetration depth of the 231 MeV ion beam (Figure 1c). The results indicate that the ions penetrate up to ≈22 µm into the device stack and glass substrate. To gain further insight into the ion–material interactions within the functional layers, an additional SRIM simulation was performed at the same ion beam energy but on a reduced scale. As shown in Figure 1d, the ion beam is distributed across all layers of the device with nearly uniform energy losses, which validates the optimal conditions for the simulation of radiation exposure.

**Figure 2:**
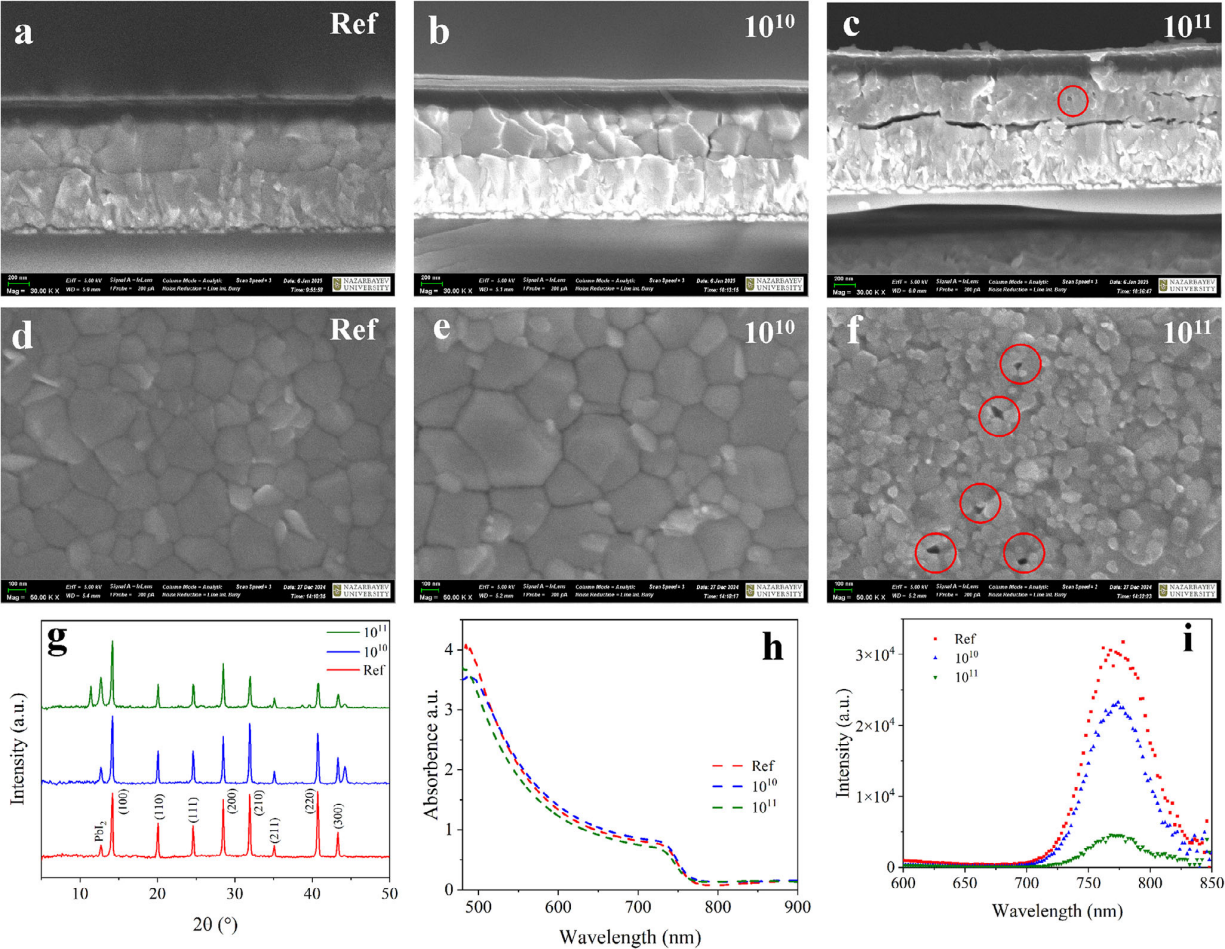
a–c) Cross‐sectional SEM images of the devices at different irradiation conditions. d–f) Top‐view SEM images, g) XRD patterns, h) optical absorption spectra, and i) PL spectra of perovskite films on glass at different irradiation conditions.

Consequently, the optical, electrical, and structural properties of the device functional layers were investigated, including the glass substrate, FTO, and MoO_3_/Au/MoO_3_ electrodes, Spiro‐OMeTAD, and perovskite layer, both before and after irradiation. The scanning electron microscope (SEM) analysis results of the fabricated device and the perovskite layer are presented in **Figure** **2**a–f. The cross‐sectional images of the device before and after irradiation with a fluence of 10^10^ nucleons cm^−2^ (Figure 2a,b) show that the structures remain largely similar, with the perovskite film thickness remaining stable and individual perovskite single crystals still distinguishable. However, when the irradiation fluence is increased to 10^11^ nucleons cm^−2^, the formation of defects (holes) distributed throughout the bulk of the perovskite layer is observed, along with a visible degradation of adhesion between the perovskite film and the substrate (Figure 2c). The surface morphology of the perovskite thin film before and after irradiation with a fluence of 10^10^ nucleons cm^−2^ remains almost unchanged: the formed perovskite crystals retain their shape and well‐defined boundaries (Figure 2d,e). In contrast, after irradiation with a fluence of 10^11^ nucleons cm^−2^, a reduction in crystal size of the perovskite layer is observed, accompanied by the appearance of defects in the form of holes on the surface of the perovskite layer (Figure 2f).

The structural properties of the complex perovskite Cs_0.05_MA_0.23_FA_0.72_Pb(I_0.77_Br_0.23_)_3_ deposited on a glass substrate were investigated before and after irradiation with xenon ions using X‐ray diffraction (XRD) spectroscopy (see Figure 2g). Prior to irradiation, the perovskite film exhibits distinct diffraction peaks at 2θ = 14.2°, 20.1°, 24.6°, 28.5°, 31.9°, 35.1°, 40.7°, and 43.3°, corresponding to the (100), (110), (111), (200), (210), (211), (220), and (300) planes of the tetragonal phase of Cs_0.05_MA_0.23_FA_0.72_Pb(I_0.77_Br_0.23_)_3_, respectively.^[^ ^33^, ^34^, ^35^ ^]^ A peak at 12.7° is also observed, attributed to the cubic phase of PbI_2_ as a secondary phase.^[^ ^14^, ^36^ ^]^ Following irradiation at a fluence of 10^10^ nucleons cm^−2^, a moderate increase in the intensity of the PbI_2_‐related peak is observed, suggesting partial decomposition of the perovskite matrix. At a higher fluence of 10^11^ nucleons cm^−2^, further deterioration of the crystalline structure is evident. The PbI_2_ peak becomes significantly more intense, and the emergence of a new diffraction maximum at 11.4° indicates the formation of the non‐photoactive δ‐phase,^[^ ^37^ ^]^ confirming phase transformations within the material.

The X‐ray photoelectron spectroscopy (XPS) results presented in Figure S1 (Supporting Information) further confirm the presence of PbI_2_, lead oxide, and structural disruption in the perovskite material after irradiation. These changes are evidenced by shifts in the Pb, I, and Br peaks and are consistent with previously reported findings.^[^ ^38^, ^39^ ^]^

The optical absorption and photoluminescence (PL) spectra of the perovskite before and after irradiation are presented in Figure 2h,i, respectively. The absorption spectra (Figure 2h) of the samples before irradiation and after exposure to a fluence of 10^10^ nucleons cm^−2^ are nearly identical, with no significant changes observed, indicating that the optical density of the material remains largely preserved at this irradiation level. However, at a higher fluence of 10^11^ nucleons cm^−2^, a noticeable decrease in optical density across the visible wavelength range is observed, likely due to the degradation of crystalline order and the formation of defect states (see Figure 2g and Figure S1, Supporting Information). Analysis of the PL spectra (Figure 2i) reveals a reduction in PL signal intensity following irradiation, which suggests the formation of structural defects and partial degradation of the perovskite material. These findings are supported by previous studies.^[^ ^40^, ^41^ ^]^ Overall, the optical measurements align well with the XRD and XPS results (Figure 2g and Figure S1, Supporting Information), confirming a consistent trend of crystal structure degradation and deterioration of optical properties with increasing irradiation fluence.

Figure S2 (Supporting Information) presents the analysis of the optical and electrical properties of the glass substrate, transparent electrodes, and Spiro‐OMeTAD before and after irradiation with 231 MeV xenon ions at varying fluences. As shown in Figure S2a (Supporting Information), the optical transmittance of the glass in the visible wavelength range decreases slightly at an irradiation fluence of 10^10^ nucleons cm^−2^. However, at a higher fluence of 10^11^ nucleons cm^−2^, a more pronounced reduction in transmittance to ≈80% is observed. Similar trends have been reported in previous studies^[^ ^42^, ^43^ ^]^ and are attributed to radiation‐induced defect formation in the glass matrix. In contrast, the Spiro‐OMeTAD layer shows no change in its spectral characteristics across all irradiation fluences; its optical transmittance remains stable (Figure S2d, Supporting Information). The transparent electrodes (FTO and MoO_3_/Au/MoO_3_) also exhibit stable transmittance at a fluence of 10^10^ nucleons cm^−2^ (Figure S2b,c, Supporting Information). However, exposure to 10^11^ nucleons cm^−2^ results in a decrease in transmittance for both electrode types, likely due to the accumulation of radiation‐induced defects in the oxide and metal layers.

The electrical properties of the electrodes, specifically sheet resistance measurements, indicate a slight increase in the resistance of the FTO layer from ≈7 to ≈9 Ω sq^−2^ after irradiation (Figure S2e, Supporting Information). In contrast, the MoO_3_/Au/MoO_3_ layer shows no measurable change in sheet resistance, remaining stable at ≈10 Ω sq^−2^ within the margin of experimental error (Figure S2f, Supporting Information). XPS analysis of the FTO and MoO_3_/Au/MoO_3_ surfaces, presented in Figure S3 (Supporting Information), reveals no significant changes in surface composition, suggesting that the electrode surfaces remain largely unaffected by the irradiation.

**Figure 3:**
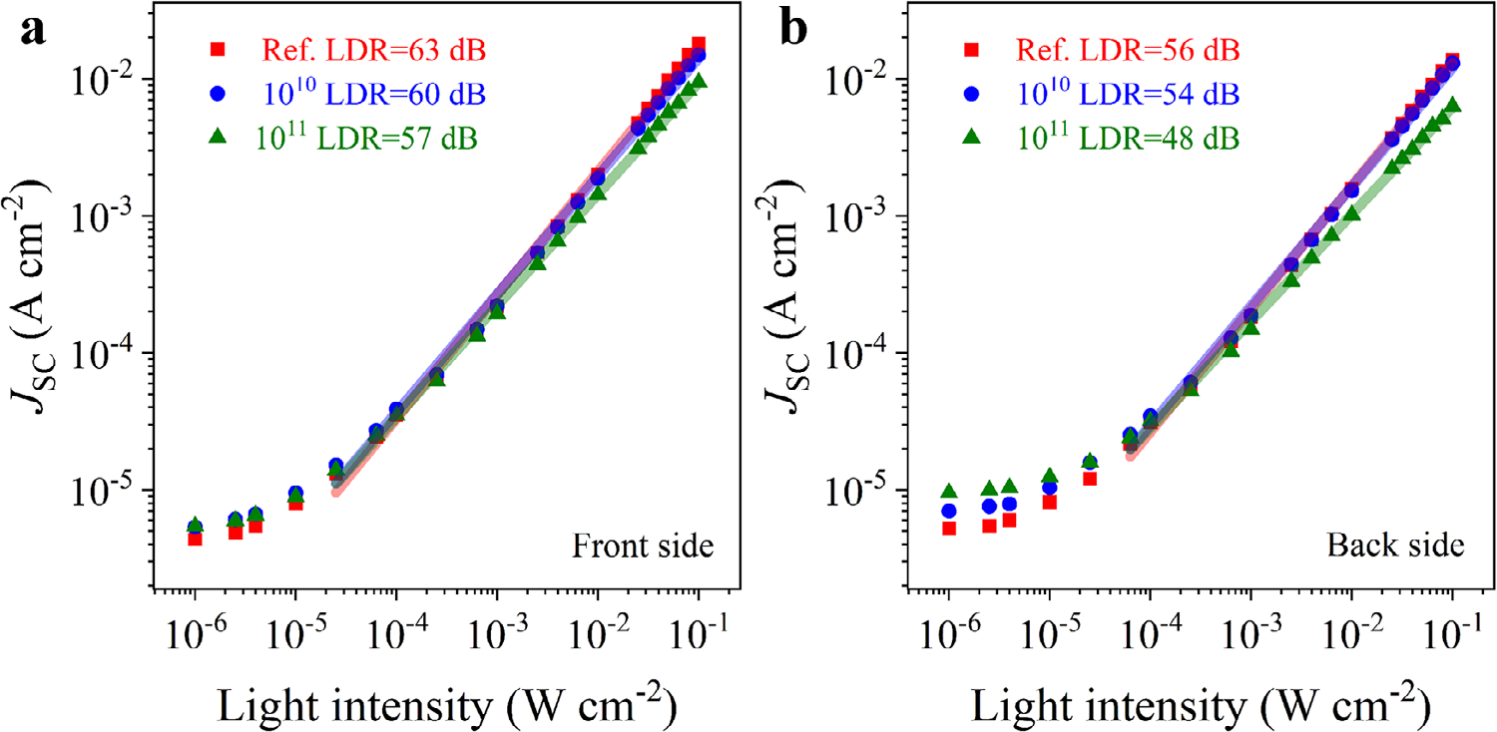
LDR of the BPPDs measured from the front‐ a) and back‐sides b) at different irradiation conditions.

A comprehensive study was conducted to evaluate the photodetector characteristics of the devices before and after irradiation. Figure S4 (Supporting Information) presents semi‐log plots of the current density‐voltage (*J‐V*) characteristics of the BPPDs, measured from both the front (glass substrate) and back (MoO_3_/Au/MoO_3_ electrode) sides under dark conditions and white light illumination at varying intensities up to 100 mW cm^−2^. The linear dynamic range (LDR) was determined for both sides of the device (**Figure** **3**) before and after irradiation, based on the range of light intensities over which the photocurrent exhibited a linear dependence on illumination power. The LDR value was calculated using the relation $LDR=20\;log\frac{J_{max}}{J_{min}}$, where *J_max_* and *J_min_* are the photocurrent densities measured at the maximum and minimum light intensities within the linear response region of the BPPDs.^[^ ^13^, ^44^ ^]^

For the front side of the device (Figure 3a), the initial LDR was ≈63 dB and decreased to ≈60 and ≈57 dB after irradiation with fluences of 10^10^ and 10^11^ nucleons cm^−2^, respectively. This decline can be attributed to the reduced optical transmittance of the glass/FTO following irradiation (see Figure S2b, Supporting Information). On the back side (Figure 3b), the initial LDR was ≈56 dB, which dropped to ≈54 dB at the lower fluence and ≈48 dB at the higher fluence. The comparatively lower LDR values observed for back‐side illumination are due to partial light absorption by the Spiro‐OMeTAD layer, particularly in the wavelength range below 400 nm (see Figure S2d, Supporting Information), which reduces the overall photocurrent. Overall, the observed decrease in LDR after irradiation is primarily associated with increased resistance in the electrodes and the formation of radiation‐induced defects in the perovskite layer.

The shunt (*R*_Sh_) and series (*R*_S_) resistances of the photodetectors were extracted from the voltage‐dependent differential resistance curves^[^ ^18^, ^45^, ^46^ ^]^ (Figure S5, Supporting Information). Following irradiation with xenon ions at different fluences, both *R*_Sh_ and *R*_S_ increased compared to the reference (as‐prepared) device. Specifically, *R*_Sh_ increased to ≈236 kΩ for both irradiation fluences, compared to ≈63 kΩ in the unirradiated device. Similarly, *R*_S_ rose from ≈24 Ω in the reference device to ≈60 Ω after low‐fluence irradiation, and exhibited a sharp increase to ≈865 Ω at the higher fluence. This significant rise in *R*_S_ is likely due to irradiation‐induced damage in the charge transport layers and electrodes (see Figure S2, Supporting Information). Additionally, the increase in series resistance may result from the formation of defects within the perovskite structure, the emergence of voids, and the degradation of adhesion between the perovskite and underlying layers in samples subjected to high‐fluence irradiation (see Figure 2).

**Figure 4:**
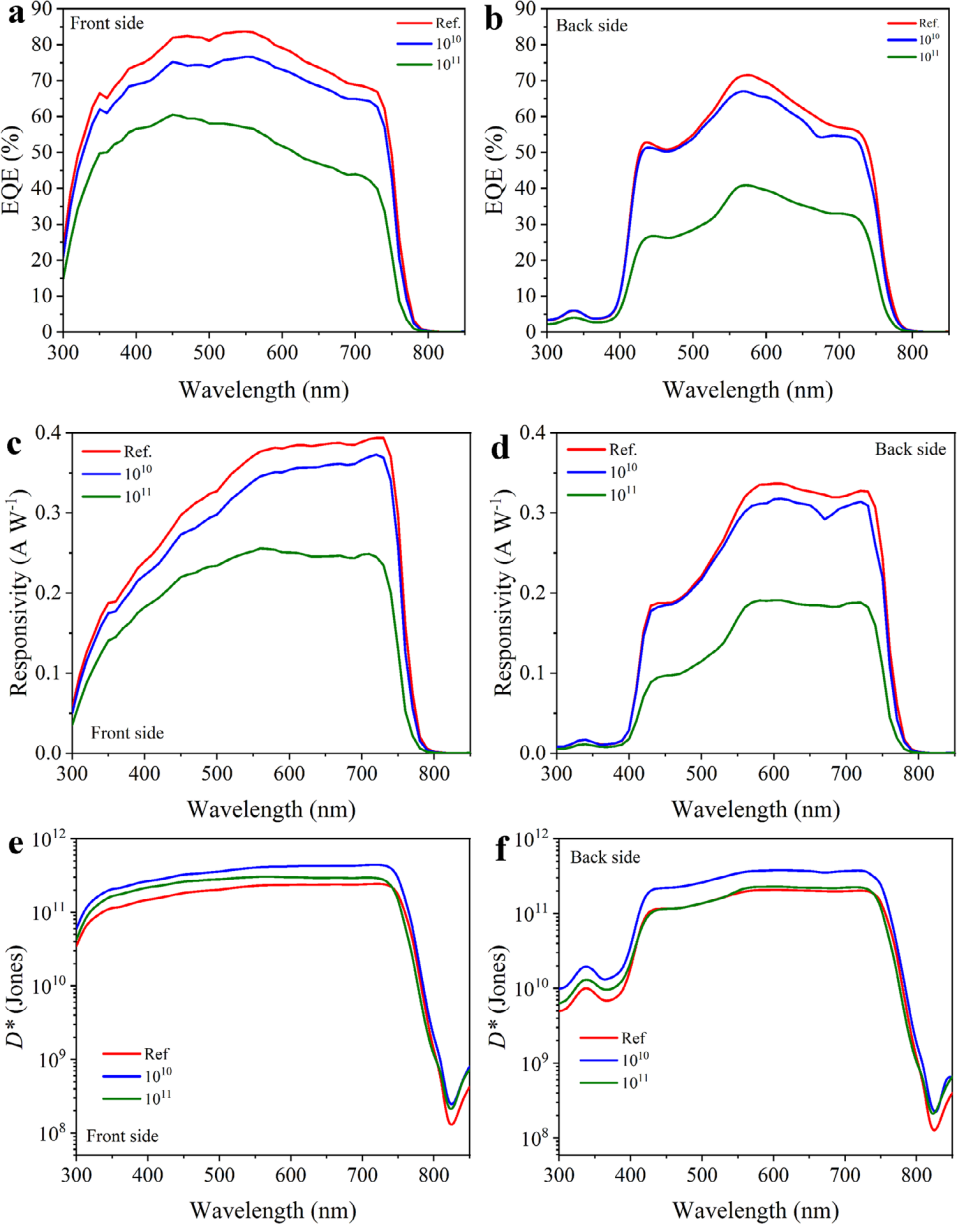
a, b) EQE, c, d) responsivity, and e, f) detectivity spectra of the BPPDs under front‐ and back‐side illumination at different irradiation conditions.

The spectral response of the photodetectors was evaluated by measuring the external quantum efficiency (EQE) as a function of wavelength under short‐circuit conditions (0 V). **Figure** **4**a,b presents the EQE spectra for front‐ and back‐side operation, respectively, measured before and after ion irradiation. For the as‐prepared devices, the front side (glass substrate) exhibits a broad photosensitive range from 300 to 750 nm, with peak EQE values exceeding 80% in the visible region (Figure 4a). In contrast, the back side (MoO_3_/Au/MoO_3_ electrode) demonstrates a slightly narrower operational range of 400 to 750 nm, with a maximum EQE of ≈70% (Figure 4b). This spectral limitation is due to the short‐wavelength cutoff introduced by the Spiro‐OMeTAD layer, which absorbs light below ≈400 nm (see Figure S2d, Supporting Information).

Following irradiation at a fluence of 10^10^ nucleons cm^−2^, the EQE values decreased slightly to ≈76% and ≈66% for the front and back sides, respectively. Upon increasing the fluence to 10^11^ nucleons cm^−2^, more substantial reductions were observed, with EQE values falling to ≈58% and ≈40% for the front and back sides, respectively. The observed decline in EQE can be attributed to two primary factors: (i) degradation of the optical and electrical properties of the BPPD functional layers (see Figure 2 and Figure S2, Supporting Information), and (ii) defect formation within the perovskite absorber as a result of ion irradiation. Additionally, the spectrally resolved bifaciality factor, calculated as the ratio of back‐side to front‐side EQE, was analyzed (Figure 4a,b). As shown in Figure S6 (Supporting Information), the reference device exhibited a high bifaciality factor of ≈90% in the 550–750 nm range. After high‐fluence irradiation, this value decreased to ≈80%, indicating a moderate reduction in back‐side performance relative to the front.

Responsivity (*R*) is a key parameter of photodiodes that quantifies the efficiency of converting incident light into an electrical signal. It is derived from the EQE using the formula $R=\frac{q\times EQE}{h\upsilon }\;$, where *q* is the elementary charge, *h* is Planck's constant, and *ν* is the frequency of the incident light.^[^ ^13^, ^22^, ^47^ ^]^ Figure 4c,d show the responsivity spectra of the as‐prepared photodetector for front‐ and back‐side illumination, respectively. The maximum responsivity values are 0.39 A W^−1^ for the front side and 0.33 A W^−1^ for the back side, both occurring at a wavelength of 730 nm. After irradiation, a gradual decline in *R* is observed. For the front side, the responsivity decreases to 0.37 A W^−1^ after low‐fluence irradiation and further to 0.25 A W^−1^ at the higher fluence. Similarly, for the back side, *R* decreases to 0.31 and 0.18 A W^−1^ at low and high fluences, respectively. This reduction reflects the degradation in device performance due to irradiation‐induced damage in the active layers and interfaces.

Another key spectral characteristic of a photodiode is the detectivity (𝐷^∗^), which quantifies the device's ability to detect weak optical signals relative to its noise level. For self‐powered photodetectors, where the noise is primarily limited by Johnson (thermal) noise, 𝐷^∗^ is calculated using the following relation: $D^{*}=R\sqrt{\frac{AR_{\mathrm{Sh}}}{4kT}}$, where *R* is the responsivity, *A* is the area of the device (0.1 cm^2^ in this study), *R*_Sh_ is the shunt resistance, *k* is the Boltzmann constant, and *T* is the absolute temperature.^[^ ^13^ ^]^ Figure 4e,f present the spectral dependence of the detectivity for the front and back sides of the BPPDs, respectively, before and after irradiation. The reference devices exhibit maximum detectivity values of 2.4 × 10^11^ Jones (front side) and 2.0 × 10^11^ Jones (back side) at a wavelength of 730 nm. Interestingly, after irradiation with a fluence of 10^10^ nucleons cm^−2^, the detectivity increases to 4.4 × 10^11^ Jones (front) and 3.7 × 10^11^ Jones (back), which is attributed to a rise in shunt resistance (see Figure S5, Supporting Information). However, upon increasing the fluence to 10^11^ nucleons cm^−2^, detectivity decreases to 2.8 × 10^11^ Jones (front side) and 2.2 × 10^11^ Jones (back side), reflecting the adverse effects of high‐fluence irradiation on charge transport and overall device performance.

**Figure 5:**
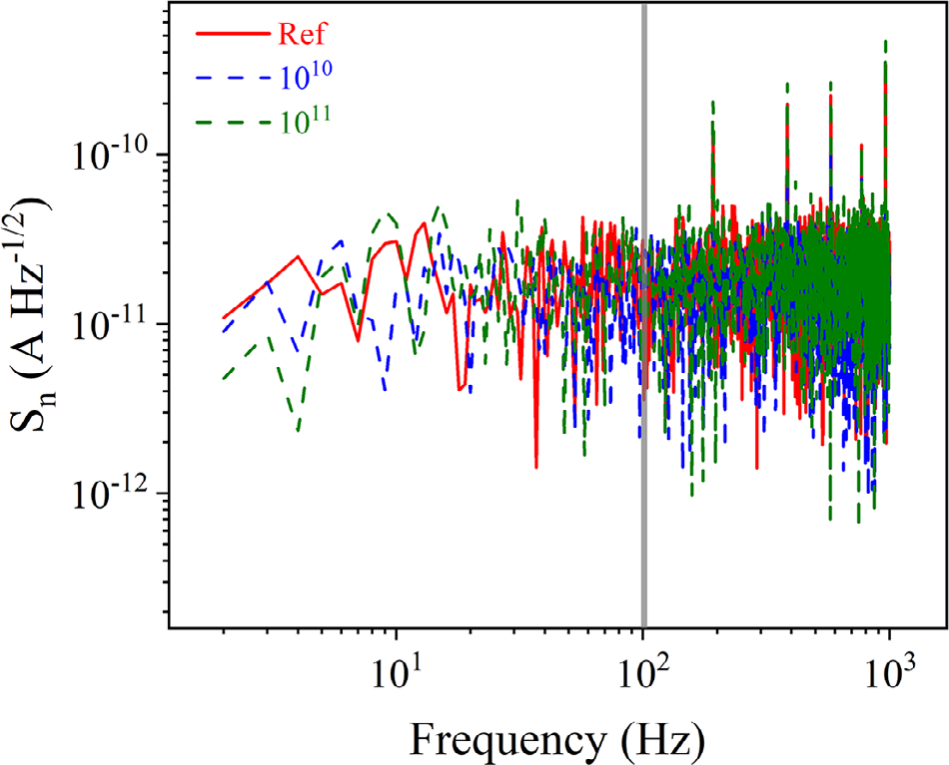
Spectral noise density of the BPPDs measured before and after irradiation.

The spectral density of the noise current (*S*_n_) is a key experimentally measured parameter of photodiodes, as it reflects the intrinsic noise level of the device. The spectral noise density was calculated following the methods described in the literature.^[^ ^13^, ^48^ ^]^ The results for the studied photodetectors are presented in **Figure** **5**. As shown, the devices exhibit a low noise current density in the pristine state: at 100 Hz, the as‐prepared photodetectors demonstrate a noise level of 3.6 × 10^−12^ A Hz^−1/2^. After low‐fluence ion irradiation, this value increases to 1.2 × 10^−11^ A Hz^−1/2^, and further rises to 2.0 × 10^−11^ A Hz^−1/2^ at the higher irradiation fluence of 10^11^ nucleons cm^−2^. The increase in noise current density is attributed to the accumulation of defects in the functional layers of the photodetector, which enhances recombination processes and introduces additional noise pathways within the device.

The specific detectivity D* of the BPPDs was calculated based on the experimentally measured spectral density of the noise current, which takes into account the combined (synergistic) contribution of all noise mechanisms, by the methods presented in the literature.^[^ ^13^, ^22^, ^48^ ^]^ Figure S7 (Supporting Information) shows the dependence of the specific detectivity of the studied BPPDs on the wavelength and frequency, both from the front and back sides, before and after ion irradiation. Analysis of the obtained graphs shows that the devices demonstrate a relatively uniform level of specific detectivity over the entire working range of wavelengths and frequencies on both sides. After low‐fluence irradiation, no significant changes are observed, indicating high radiation resistance of the material at moderate irradiation levels. However, when the fluence is increased to high values, a noticeable decrease in the specific detectivity is recorded, which may be due to the degradation of the optical and electrical characteristics of the functional layers and due to the formation of defects in the perovskite layer.

**Figure 6:**
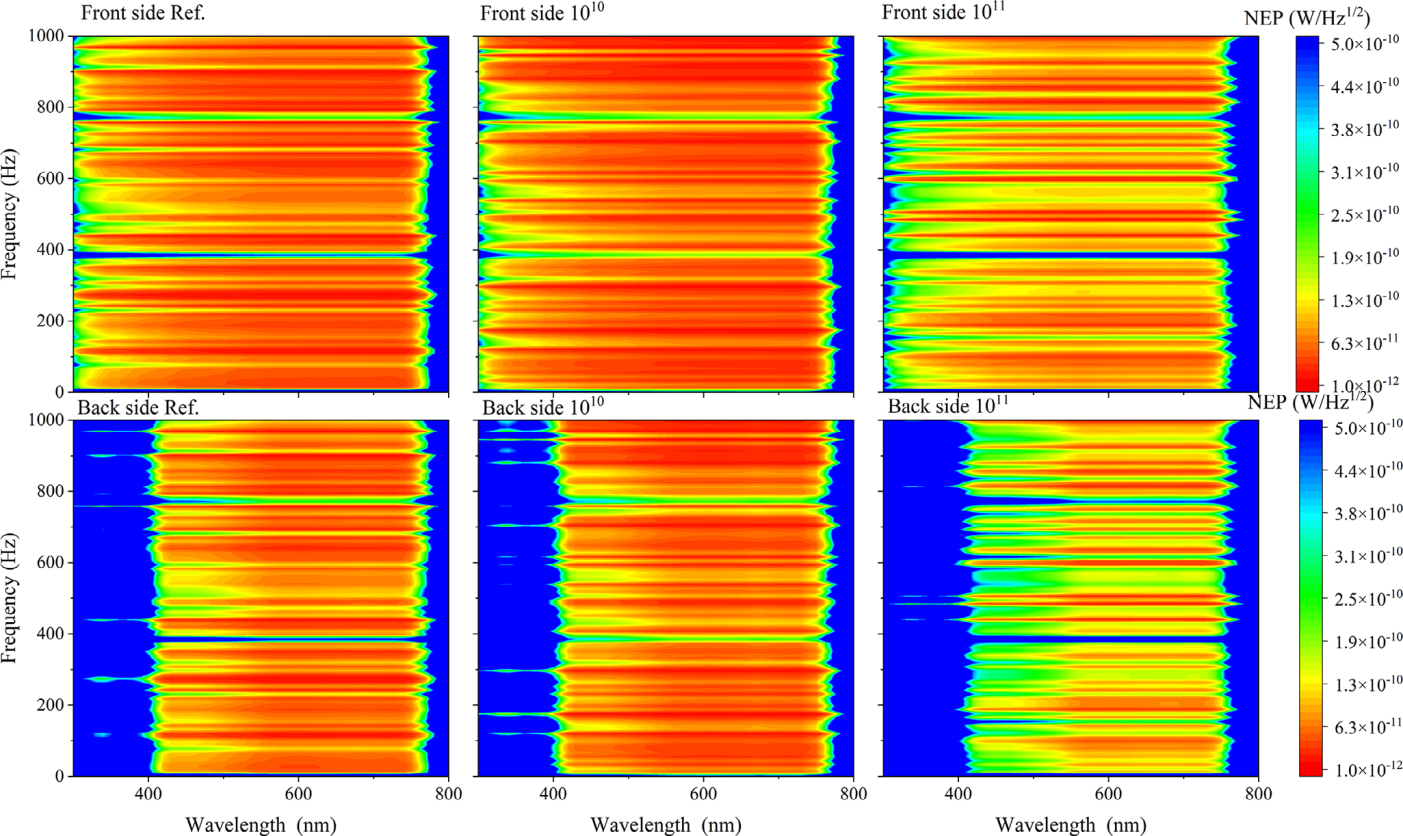
*NEP* of the BPPDs for front‐ a–c) and back‐side d–f) illumination: (a, d) before irradiation, (b, e) after irradiation at a fluence of 10^10^ nucleons cm^−2^, and (c, f) after irradiation at 10^11^ nucleons cm^−2^.

The noise equivalent power (*NEP*) is a widely used parameter that quantitatively characterizes the sensitivity of a photodetector. It represents the minimum optical signal power required to achieve a signal‐to‐noise ratio of unity within a 1 Hz bandwidth. *NEP* is calculated using the relation: $NEP=\frac{S_{n}}{R}\;$. The *NEP* values were calculated based on the experimentally measured noise current spectral density (see Figure 5) and the wavelength‐dependent responsivity (Figure 4c,d). The resulting *NEP* distributions are shown as 3D plots in **Figure** **6**, mapped over frequency and wavelength coordinates. From the plots, it is evident that the reference photodetector exhibits low *NEP* values (< 10^−12^ W Hz^−1/2^) over a broad spectral range: from 300 to 750 nm for front‐side illumination and from 400 to 750 nm for back‐side illumination (Figure 6a,d). After low‐fluence irradiation, no significant change in *NEP* is observed (Figure 6b,e), indicating preserved sensitivity. However, at higher irradiation fluences, the device performance on both sides deteriorates, as reflected by a noticeable increase in *NEP* values (Figure 6c,f). This increase signifies a decline in photodetector sensitivity, which is attributed to radiation‐induced degradation of the active layers and the accumulation of defect states.

The time response of the photodetectors was evaluated by measuring the photocurrent as a function of time, with the response time defined as *t_res_* = *t_rise_*  + *t_fall_*. Figure S8 (Supporting Information) displays the time response curves for the as‐prepared device (a), and for devices irradiated with xenon ions at fluences of 10^10^ nucleons cm^−2^ (b) and 10^11^ nucleons cm^−2^ (c). Measurements were performed under pulsed illumination from both the front and back sides of the devices. The experimental methodology and analysis procedures are described in detail in previous works.^[^ ^13^, ^48^ ^]^ For the as‐prepared device (Figure S8a, Supporting Information), the rise time (*t_rise_*) is ≈25.9 ms (front side) and 6.8 ms (back side), while the fall time (*t_fall_*) is equally short at ≈1.6 ms for both, indicating fast photoresponse and recovery.

After irradiation with a fluence of 10^10^ nucleons cm^−2^ (Figure S8b, Supporting Information), a noticeable increase in *t_rise_* is observed, rising to 74.5 ms (front) and 10.6 ms (back) – likely due to the formation of trap states in the perovskite layer and degradation of charge transport pathways. However, *t_fall_* remains nearly unchanged at ≈1.6 ms. With further irradiation at a fluence of 10^11^ nucleons cm^−2^ (Figure S8c, Supporting Information), a dramatic slowdown in photoresponse occurs: *t_rise_* increases to 9.9 s (front) and 9.6 s (back), while *t_fall_* also increasing significantly, reaching 0.3 s on the back side. These effects are attributed to severe crystal structure degradation and a high density of trap states induced by ion exposure. Overall, the time response behavior of the devices under irradiation reflects a typical trend in perovskite‐based photodetectors – namely, a progressive slowdown of charge transfer processes with increasing radiation fluence. These findings are consistent with previous reports on radiation effects in perovskite optoelectronics.^[^ ^13^ ^]^ The key photodetector characteristics of the devices with calculated standard deviation before and after irradiation are summarized in Table S1 (Supporting Information).

The steady‐state open‐circuit voltage (*V_oc_*) versus light intensity (*I*) measurements were used to identify the dominant recombination pathways in the devices to elucidate the relationship between irradiation‐induced defects and device performance. The slope of the *V_oc_* versus *ln(I)* (see Figure S9a Supporting Information) reflects the prevailing recombination mechanism.^[^ ^49^, ^50^, ^51^ ^]^ The reference device exhibited a slope of 1.2* kT/q*, slightly higher than *kT/q*, suggesting a minor contribution from trap‐assisted Shockley‐Read‐Hall (SRH) recombination in the perovskite bulk. After low‐fluence xenon ion exposure (10^10^ nucleons cm^−2^), the slope increased to 1.85 kT/q, and further to 2.08 kT/q at higher fluence (10^11^ nucleons cm^−2^), confirming that SRH recombination becomes the dominant loss mechanism due to the formation of deep trap states.^[^ ^51^, ^52^ ^]^ These results are consistent with the PL spectra of the irradiated perovskite films (see Figure 2i).

*V_oc_* decay was analyzed to further quantify recombination dynamics (see Figure S9b Supporting Information). Assuming *n ≈ exp(qV_oc_/kT)*, carrier lifetimes (*τ*) were compared at equivalent *V_oc_* values corresponding to similar nonequilibrium carrier densities.^[^ ^53^, ^54^, ^55^ ^]^ The *τ* at the *V_oc_* = 0.84 V decreased from 3 × 10^−5^ s (for the reference) to 2.3 × 10^−5^ and 1 × 10^−5^ s after low‐ and high‐fluence irradiation, respectively, indicating accelerated carrier recombination caused by ion‐induced defects. Next, the *τ* were fitted within a multi‐mechanism recombination model,^[^ ^14^, ^56^, ^57^, ^58^ ^]^ enabling the extraction of coefficients corresponding to bulk and surface trap‐assisted processes. Both coefficients increased significantly with irradiation fluence, confirming higher defect densities in the perovskite bulk and at the ETL/perovskite and HTL/perovskite interfaces (see Figure S10 Supporting Information).

The observed degradation in the main photodetector parameters at high fluence can be attributed to increased SRH recombination resulting from irradiation‐induced deep‐level traps. The formation of Pb and I vacancies and Pb‐I antisite defects introduces band gap states that facilitate nonradiative recombination centers and hinder charge extraction. This enhanced SRH process leads to reduced carrier lifetime and mobility, consistent with the slower photoresponse and decreased EQE and responsivity observed experimentally. While our data clearly indicate the increase in trap‐assisted recombination, a detailed quantitative analysis of trap energy levels and their densities requires additional material characterization.

The experimental results demonstrate that the BPPDs possess outstanding radiation resistance under 231 MeV xenon ion irradiation at fluences of 10^10^–10^11^  nucleons cm^−2^. For comparison, Table S2 (Supporting Information) summarizes the responsivity and detectivity of the photodetectors based on perovskite and compound semiconductor before and after ionizing irradiation, highlighting the superior stability of our devices under xenon‐ion exposure. It is important to note that light particles such as protons mainly induce ionization and shallow point defects, leading to moderate, reversible degradation in device performance.^[^ ^7^, ^8^, ^21^ ^]^ In contrast, heavy ions deposit far greater energy via nuclear collisions, causing intense atomic displacement cascades and producing complex vacancy clusters and amorphous regions within the perovskite and transport layers.^[^ ^59^, ^60^ ^]^ This combination of strong displacement and ionization damage leads to deeper, more stable trap formation and partial phase decomposition. Overall, the excellent radiation tolerance under xenon ion irradiation combined with the bifacial architecture makes the investigated photodetectors strong candidates for the next generation of optoelectronic systems designed for reliable operation under harsh radiation environments.

## 3 Conclusion

In summary, we systematically investigated the robustness of high‐performance bifacial perovskite photodetectors (BPPDs) under 231 MeV xenon ion irradiation at fluences of 10^10^–10^11^ nucleons cm^−2^. The devices exhibited excellent initial performance, with EQE ≥ 80% under front‐side illumination, broad spectral responsivity *R* ≈ 0.39 A W^−1^), and high specific detectivity (𝐷^∗^ ≅ 2.4 × 10^11^ Jones at 730 nm). Back‐side performance was limited at short wavelengths due to absorption by the Spiro‐OMeTAD layer. Low‐fluence irradiation (10^10^ nucleons cm^−2^) caused minimal degradation, with photoresponse characteristics remaining stable and detectivity slightly improving due to an increase in shunt resistance. At higher fluence (10^11^ nucleons cm^−2^), partial loss of perovskite crystallinity, trap‐state formation, and emergence of PbI_2_ phases were observed, alongside increased series resistance and optical deterioration of transparent electrodes. These changes reduced EQE, responsivity, and detectivity by ≈30–40% for both illumination directions and slowed photoresponse dynamics from milliseconds to seconds, indicating hindered charge transport. Even so, the devices retained over 60% of their initial performance and maintained low spectral noise density across the full wavelength range, emphasizing their notable radiation tolerance.

Looking forward, our findings highlight several pathways for further improving stability at high ion fluence. Materials engineering approaches, such as incorporating defect‐tolerant perovskite compositions and more radiation‐hardened transport layers, could mitigate damage induced by dense ion tracks. Interface and passivation strategies may suppress defect propagation and reduce recombination losses, while device architecture optimizations, including buffer or protective layers and improved transparent electrodes, could enhance robustness against heavy‐ion exposure. These directions provide a roadmap toward realizing perovskite photodetectors with sustained performance under extreme radiation conditions. Overall, this work demonstrates that BPPDs are promising candidates for optoelectronic applications in space and other harsh environments, while also identifying key strategies for advancing radiation‐hardened perovskite technologies.

## 4 Experimental Section

### Materials

All chemicals were used without any further purification. Lead iodide (PbI_2_, 99%), lead bromide (PbBr_2_, 99.999%) chlorobenzene (CB, 99.80%), bis(trifluoromethane)sulfonimide lithium salt (Li‐TFSI, 99%), 4‐tert‐Butylpyridine (4‐tBP, 98%), cesium iodide (CsI, 99.999%), acetone (99.80%), dimethylformamide (DMF) and dimethyl sulfoxide (DMSO) were obtained from Merck. Methylammonium iodide (MAI, 99.995%) and Formamidinium iodide (FAI, >99.99%) were obtained from GreatCell Energy. 2‐Propanol (IPA, 99.80%), was obtained from a local supplier. 2,2′,7,7′‐Tetrakis‐(N,N‐di‐4‐methoxyphenylamino)‐9,9′‐spirobifluorene (Spiro‐MeOTAD, 99.50%) was obtained from Lumtec. Fluorine‐doped tin oxide (FTO) glass (glass ≈2.2 mm and FTO ≈500 nm; sheet resistance: ≈7 Ω sq^−1^) was purchased from OPVTech. Gold (Au, 99.99%) and molybdenum oxide (MoO_3_, 99.95%) were obtained from Kurt J. Lesker. Tin(IV) oxide colloidal nanoparticle solution (SnO_2_, 15 wt.% in H_2_O colloidal dispersion) was purchased from Alfa Aesar.

### Device Fabrication

To fabricate bifacial perovskite photodetectors, FTO glass substrates were sequentially cleaned in an ultrasonic bath for 10 min each in deionized (DI) water with detergent, DI water, acetone, and IPA, then dried using a nitrogen gun. The cleaned substrates were subsequently treated with UV‐ozone for 15 min to enhance surface wettability.

The tin oxide electron transport layer (ETL) was prepared from a commercial colloidal SnO_2_ precursor solution, which was diluted with DI water at a volume ratio of 1:3. The SnO_2_ layer was deposited via spin coating at 4000 rpm for 30 s and annealed at 150 °C on a hot plate for 30 min. After annealing, the substrates underwent an additional UV‐ozone treatment for 25 min to further improve film quality and interfacial properties.

The hybrid triple‐cation perovskite ink, Cs_0.05_MA_0.23_FA_0.72_Pb(I_0.77_Br_0.23_)_3_, was prepared according to the procedure described by Saliba et al.^[^ ^61^ ^]^ The perovskite layer was deposited inside a nitrogen‐filled glovebox using a two‐step spin‐coating process: first at 2000 rpm (ramp rate: 200 rpm s^−1^) for 10 s, followed by 4000 rpm (ramp rate: 2000 rpm s^−1^) for 30 s. Ten seconds before the end of the second step, 200 µL of chlorobenzene was dynamically dropped onto the spinning substrate as an anti‐solvent. The films were then annealed on a hot plate at 100 °C for 10 min and allowed to cool to room temperature.

Next, the hole transport layer (HTL) was deposited by spin coating a Spiro‐OMeTAD solution in chlorobenzene (72.5 mg mL^−1^ Spiro‐OMeTAD with 30 µL 4‐tert‐butylpyridine and 17.5 µL LiTFSI solution, 520 mg mL^−1^ in acetonitrile). Finally, the DMD (dielectric‐metal‐dielectric) top contact was deposited via thermal evaporation (Angstrom Engineering Inc., Kitchener, Canada) under a base pressure of 2 × 10^−6^ Torr. Deposition rates were set to 0.5 Å s^−1^ for MoO_3_ and 0.1 Å s^−1^ for Au. The optimal thicknesses for the MoO_3_/Au/MoO_3_ stack were 10/10/35 nm, respectively.

### Irradiation

Irradiation of the samples with xenon ion beams was carried out at the DC‐60 heavy ion accelerator of the Astana branch of the Institute of Nuclear Physics. Beams of ^132^Xe^22+^ ions were produced by the DECRIS‐3 ECR ion source and subsequently accelerated in the cyclotron's magnetic resonance system to an energy of 1.75 MeV nucleon^−1^ (total energy of 231 MeV). The irradiation with ion beams was performed on channel No.3 of the accelerator. The samples were mounted on a water‐cooled (18 °C) target holder, with a magnetic electron suppression system placed in front of the targets. To ensure uniform irradiation of the samples in channel No. 3, an electromagnetic beam scanning system was used. This system provides irradiation of a stationary target with dimensions of 50 × 50 mm^2^ with a particle distribution uniformity across the irradiated area better than 5%. The irradiation chamber was equipped with a pumping system using forevacuum and turbomolecular pumps. During irradiation, the vacuum in the chamber was maintained below 5 × 10^−6^ Torr.

In this study, the particle flux (dose rate) was carefully controlled and maintained at 5.75 × 10^7^ cm^−2^ s^−1^ with a constant beam current of 3.05 × 10^−9^ A throughout the irradiation process. The detailed irradiation parameters are summarized in Table S3 (Supporting Information). These controlled conditions were selected to eliminate the influence of varying defect accumulation rates and their subsequent evolution within the damaged layer – factors that require strict control in irradiation studies. As demonstrated in previous work ^[^ ^62^ ^],^ variations in flux during heavy‐ion irradiation can significantly alter the degree of structural disorder at identical fluence levels due to differences in defect generation and evolution dynamics. By maintaining a constant flux and beam current in the present experiment, such effects were effectively excluded, enabling direct comparison of the results across different fluences and allowing reliable assessment of defect accumulation kinetics in the irradiated samples. In general, lower dose rates allow more time for self‐healing and dynamic annealing of radiation‐induced defects,^[^ ^7^, ^21^ ^]^ resulting in reduced defect density and slower degradation, whereas higher dose rates promote the rapid accumulation of displacement damage, often leading to enhanced structural disorder and faster performance deterioration. Studies on proton‐irradiated perovskite devices have shown that lower flux conditions tend to preserve optoelectronic performance more effectively due to concurrent defect relaxation processes.^[^ ^7^, ^14^ ^]^

### Materials Characterizations and Device Measurements

An ultraviolet‐visible spectrometer (UV–Vis, Lambda 1050, PerkinElmer, USA) was employed to analyze the optical properties of thin films. X‐ray diffraction (XRD, Rigaku Smartlab, Japan) analysis was used to study the structural properties of perovskite layers. The photoluminescence (PL) spectra of samples were obtained using a FLS 1000 spectrometer (Edinburgh Instruments, UK). The top morphology of perovskite and cross‐section images of device functional layers were obtained using a scanning electron microscope (SEM, Crossbeam 540, Zeiss, Germany). The sheet resistance of electrodes was measured using a four‐point system (RM3000, Jandel, UK). The chemical bonds of thin films were investigated using an X‐ray photoelectron spectrometer with a monochromatic X‐ray source of Al K_α_ at 1486.6 eV (NEXSA, Thermo Scientific).

A semiconductor device analyzer (B1500A, Keysight, USA) coupled with a 3A+ solar simulator (Oriel Sol3A, Newport, USA) providing an AM 1.5G illumination intensity of 100 mW cm^−2^ was used to measure the *J–V* characteristics of the devices. The linear dynamic range (LDR) was determined by recording *J‐V* curves under varying light intensities, adjusted using a set of neutral density optical filters (NEK01S, Thorlabs, USA). An external quantum efficiency (EQE) measurement system (ORIEL IQE 200, Newport, USA) was used to acquire the EQE spectra of the photodetectors. For the noise current measurements, a battery‐powered low‐noise current preamplifier (SR570, Stanford Research Systems, USA) combined with an oscilloscope (SDS5032X, Siglent, China) operating in Fast Fourier Transform (FFT) mode was employed inside a shielded Faraday cage to minimize external interference. Chronoamperometry measurements were performed using a potentiostat (Autolab PGSTAT302N, Metrohm, Switzerland) to evaluate the photoresponse time and cutoff frequency of the devices.

### Funding

This research was supported by scientific research grants from the Ministry of Science and Higher Education of the Republic of Kazakhstan (Grant Nos. AP23483937, AP19576154, and BR28712419) and by the Nazarbayev University Collaborative Research Program (Grant No. 211123CRP1613). H.P. gratefully acknowledges support from the Polish National Agency for Academic Exchange through the Ulam NAWA Program (Grant No. BNI/ULM/2024/1/00019).

## Conflict of Interest

The authors declare no conflict of interest.

## Supporting information



Supporting Information

## Data Availability

The data that support the findings of this study are available from the corresponding author upon reasonable request.
