# Supplementary material for: Evaluating the Resistance of Bifacial Perovskite Photodetectors to Xenon Ion Irradiation
Source: Adv Sci (Weinh). 2025 Nov 9;13(9):e16418. doi: 10.1002/advs.202516418 (PMC12904014; doi:10.1002/advs.202516418)
Supplement: Supplementary file 1 — Supporting Information [file ADVS-13-e16418-s001.docx]

Supporting Information

Evaluating the Resistance of Bifacial Perovskite Photodetectors to Xenon Ion Irradiation

Yerassyl Yerlanuly, Hryhorii P. Parkhomenko, Almaz R. Beisenbayev, Maxim V. Zdorovets, Annie Ng, and Askhat N. Jumabekov*

Y. Yerlanuly and A. N. Jumabekov

Department of Physics, School of Sciences and Humanities, Nazarbayev University, Astana 010000, Kazakhstan

E-mail: [askhat.jumabekov@nu.edu.kz](mailto:askhat.jumabekov@nu.edu.kz)

Y. Yerlanuly

Kazakh-British Technical University, Almaty 050000, Kazakhstan

H. P. Parkhomenko

Faculty of Physics and Astronomy, Adam Mickiewicz University, Poznań 61-614, Poland

A. R. Beisenbayev

Department of Chemical and Materials Engineering, School of Engineering and Digital Sciences, Nazarbayev University, Astana 010000, Kazakhstan

M. V. Zdorovets

The Institute of Nuclear Physics, Almaty 050032, Kazakhstan

A. Ng

Department of Electrical and Computer Engineering, School of Engineering and Digital Sciences, Nazarbayev University, Astana 010000, Kazakhstan


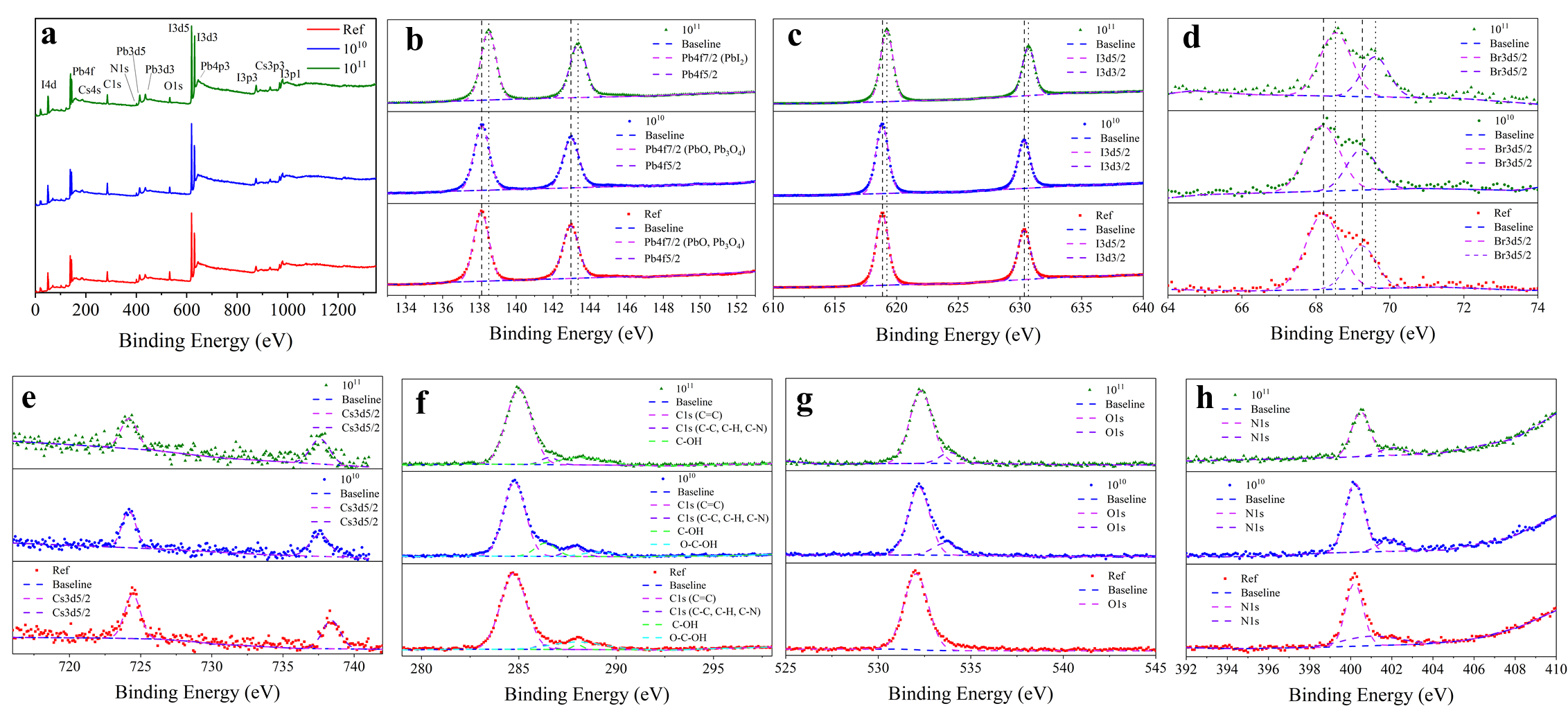


Figure S1. XPS analysis of perovskite surface before and after irradiation


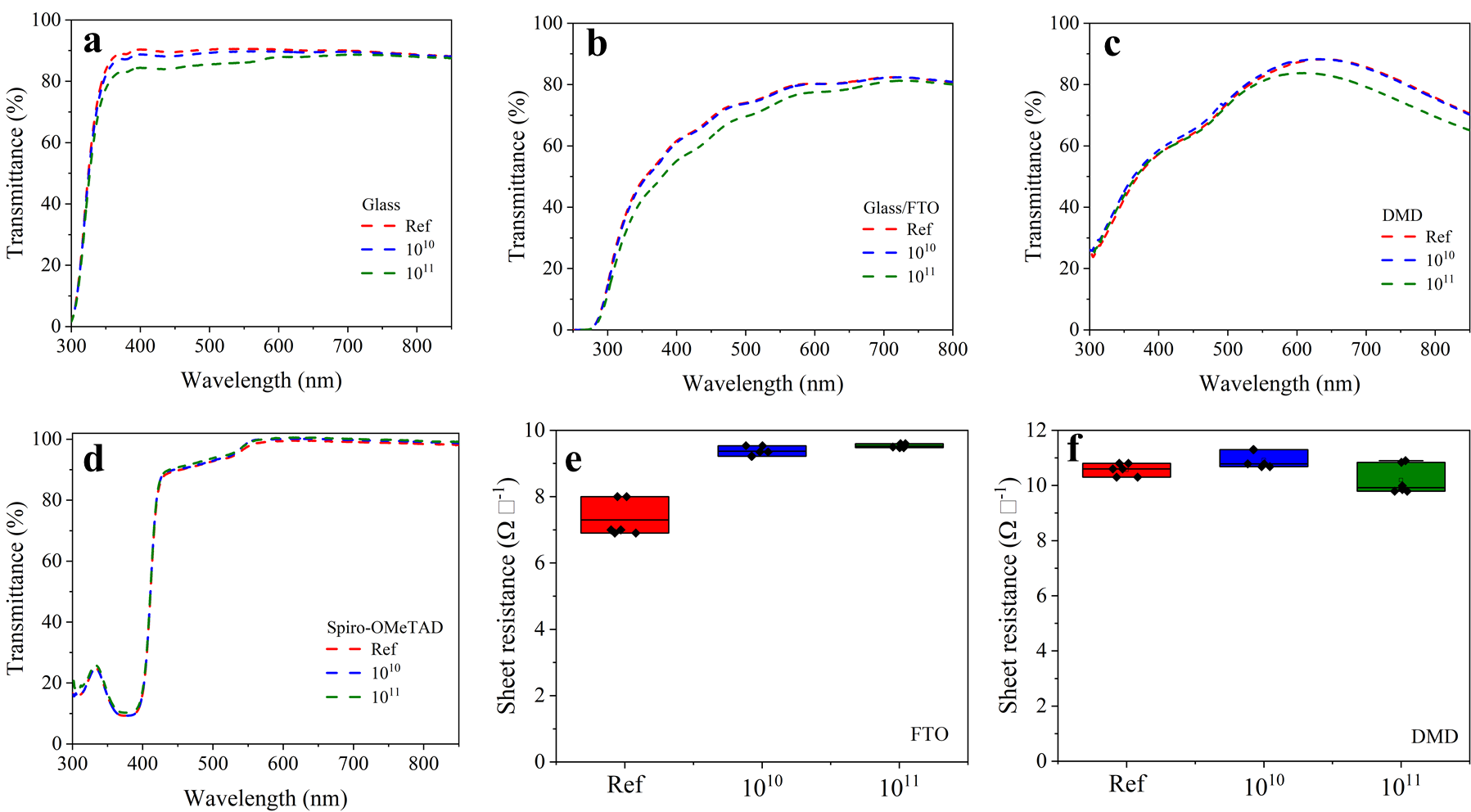


Figure S2. Optical and electrical characteristics of the materials used in the BPPDs before and after irradiation at different doses. (a–d) Optical transmittance spectra of glass, fluorine-doped tin oxide (FTO), MoO_3_/Au/MoO_3_ dielectri/metal/dielectric (DMD) trilayer, and Spiro-OMeTAD, respectively. (e–f) Sheet resistance of FTO and DMD, respectively, measured before and after irradiation.


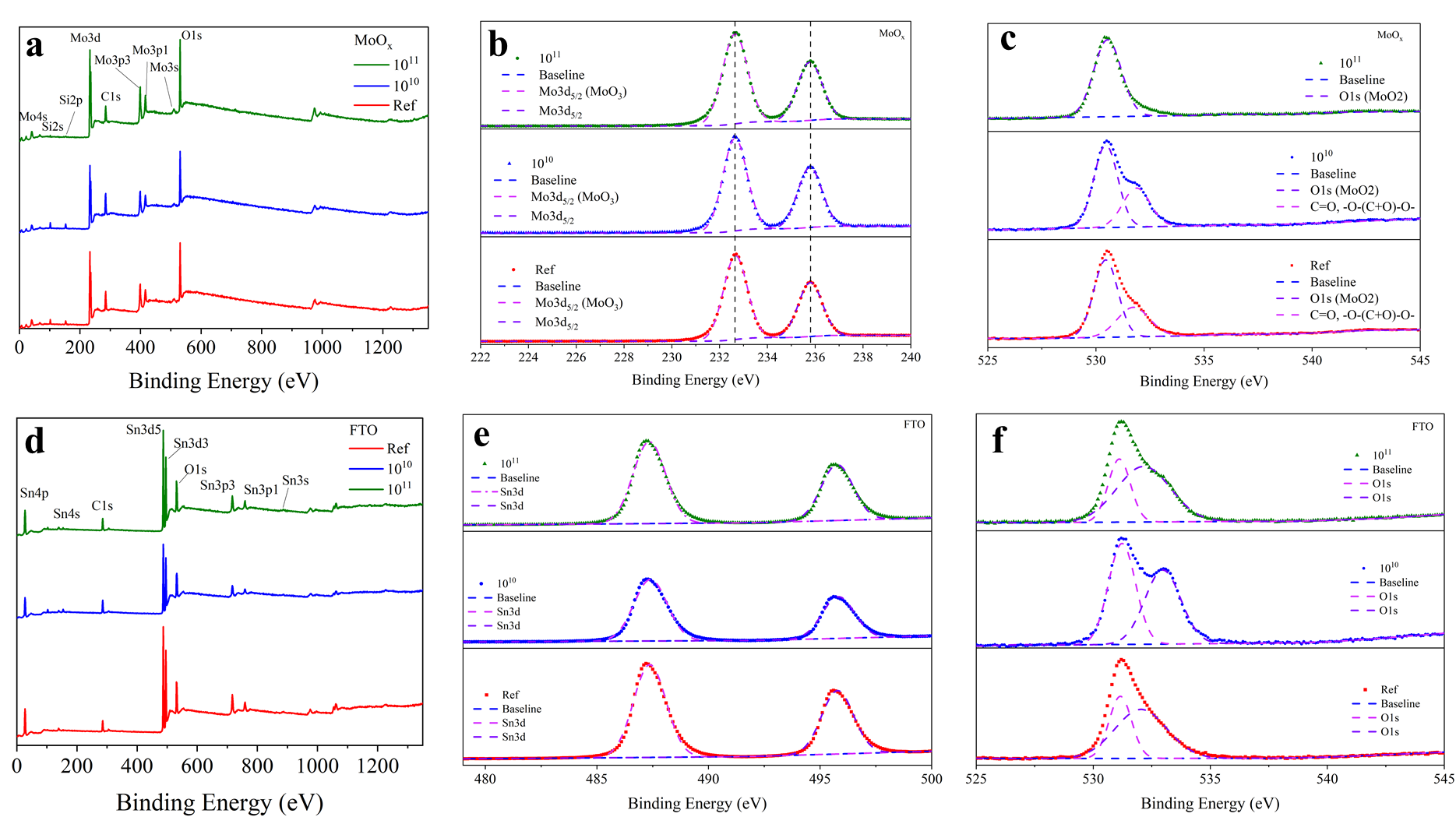


Figure S3. XPS analysis of the electrode surface before and after irradiation. (a-c) FTO and (d-f) DMD


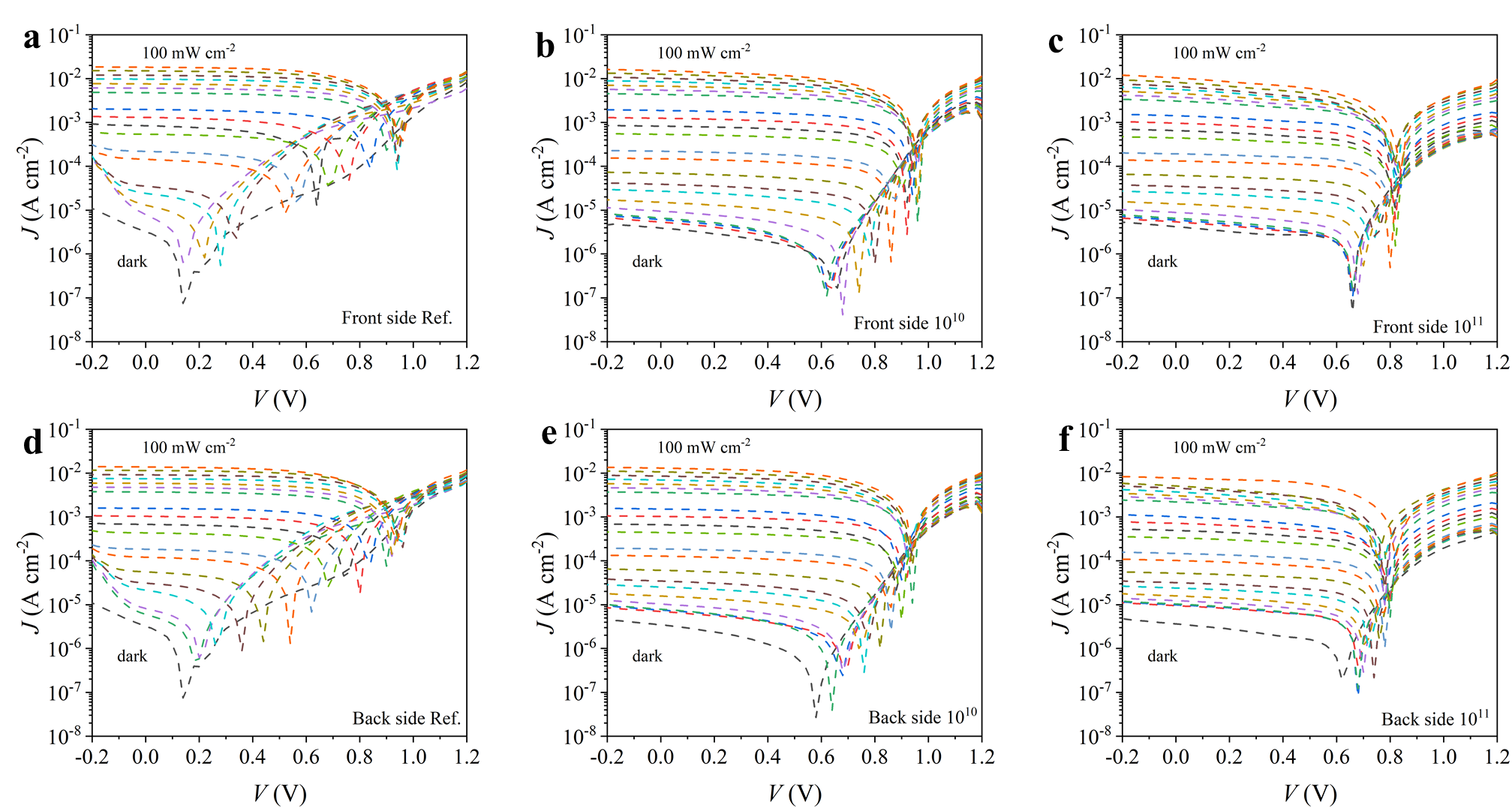


Figure S4. Dark and light *J-V* characteristics of devices from the front (a–c) and back (d–f) sides before (a, d) and after (b–f) irradiation.


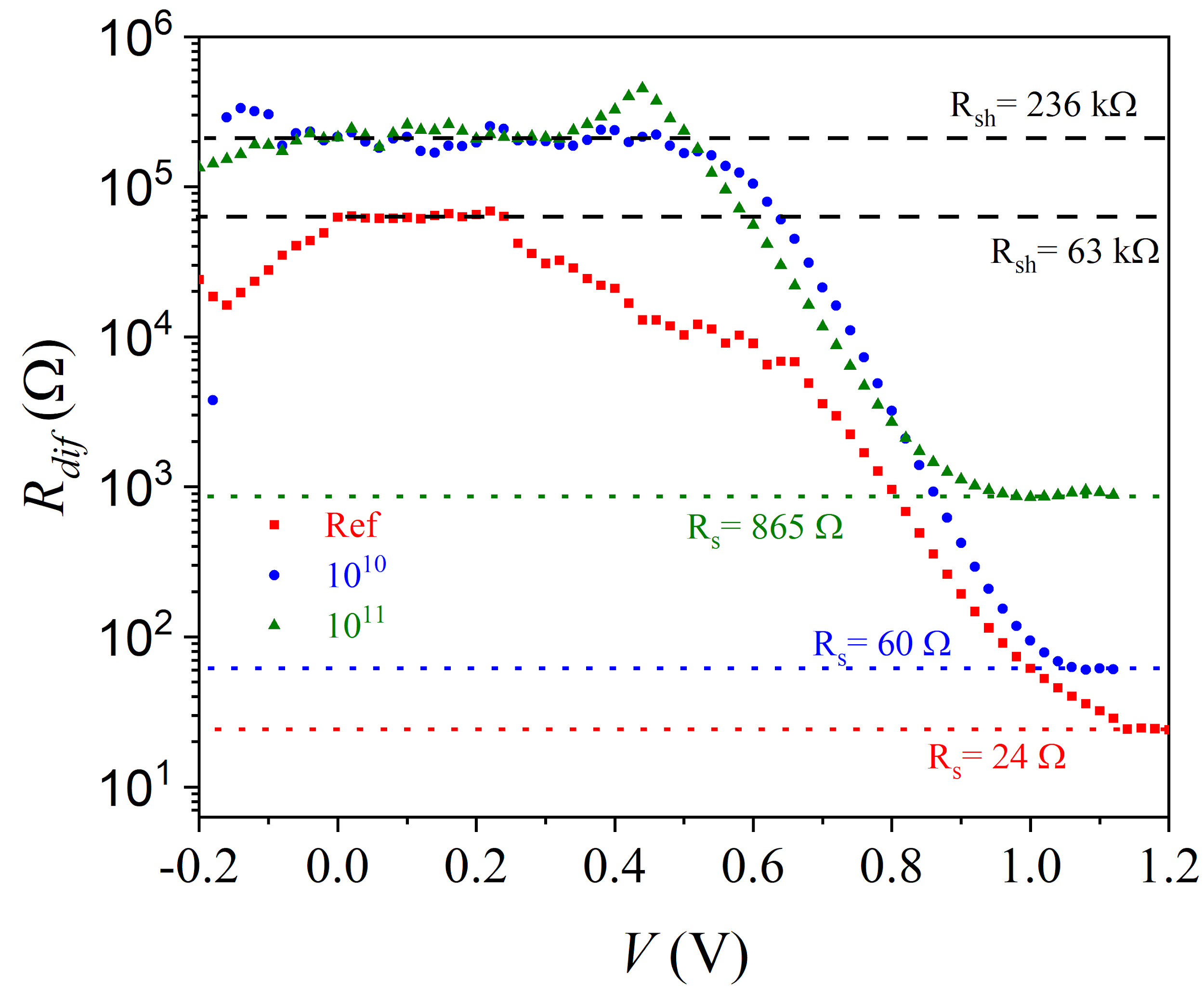


Figure S5. Dependence of the differential resistance on the applied bias of the perovskite devices before and after irradiation


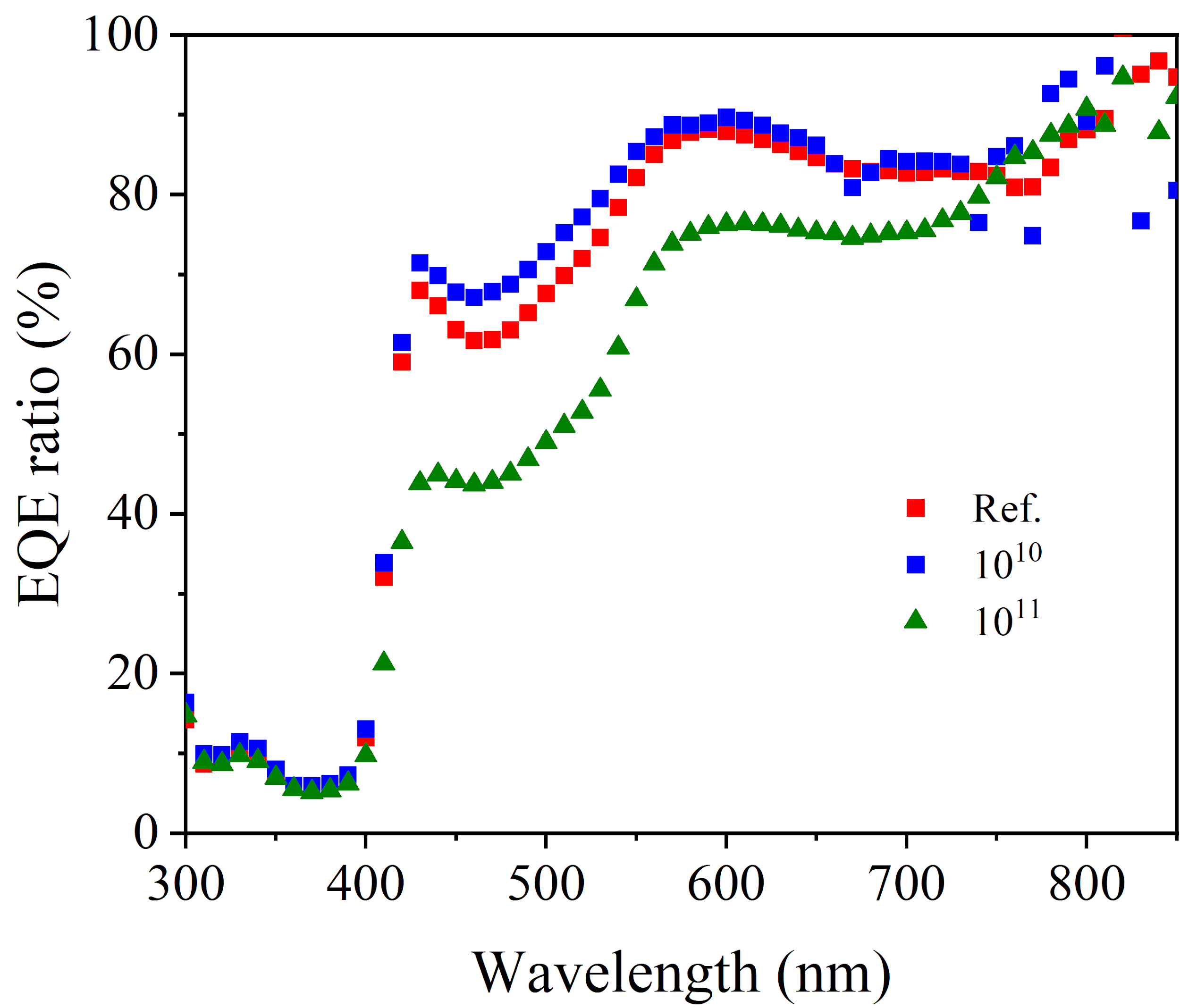


Figure S6. Ratio of the front- and back-side illumination EQEs for each wavelength.


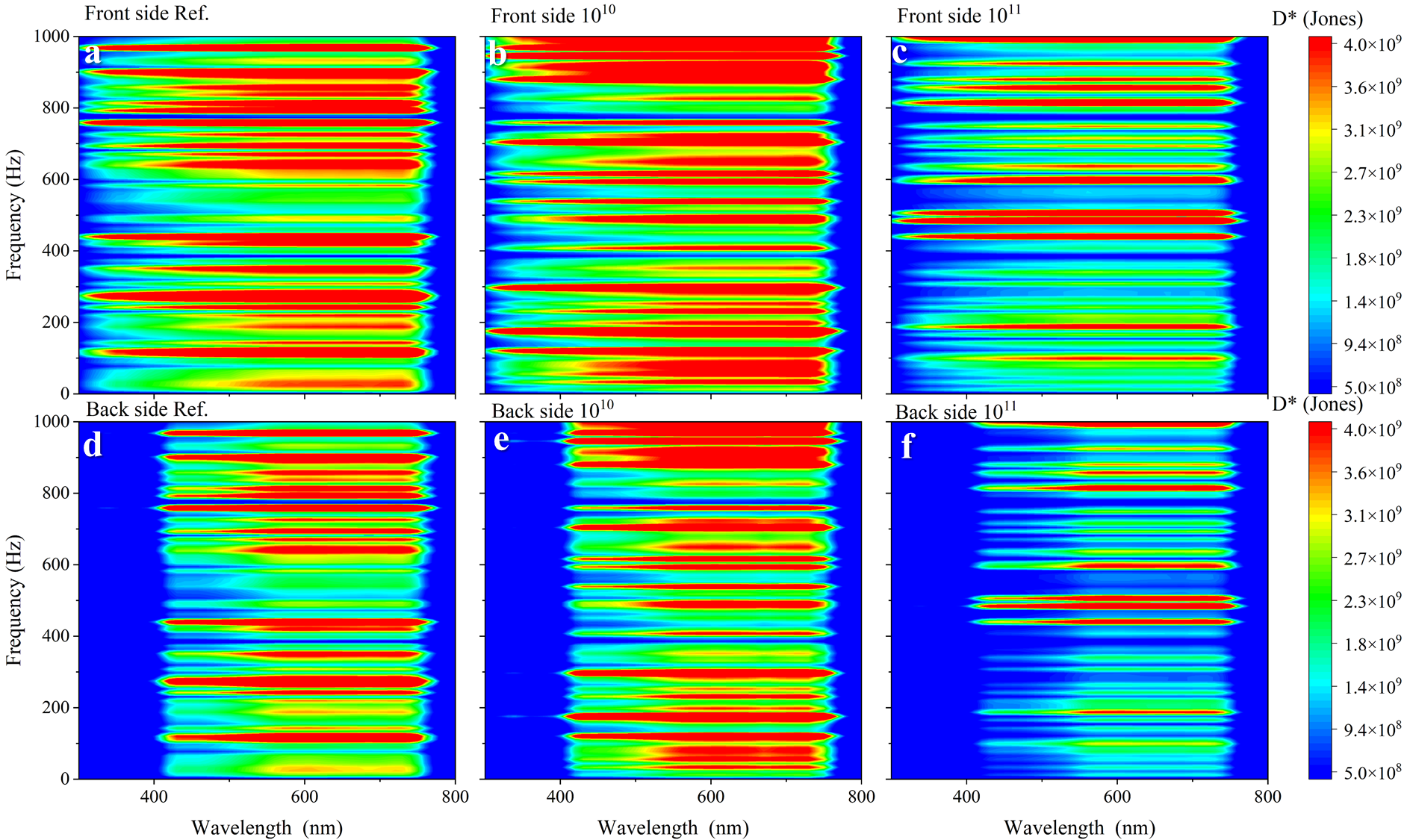


Figure S7. *D** for the perovskite photodiodes as a function of frequency and wavelength: front- (a–c) and back-side (d–f) illumination before (a, d) and after (b–f) irradiation.


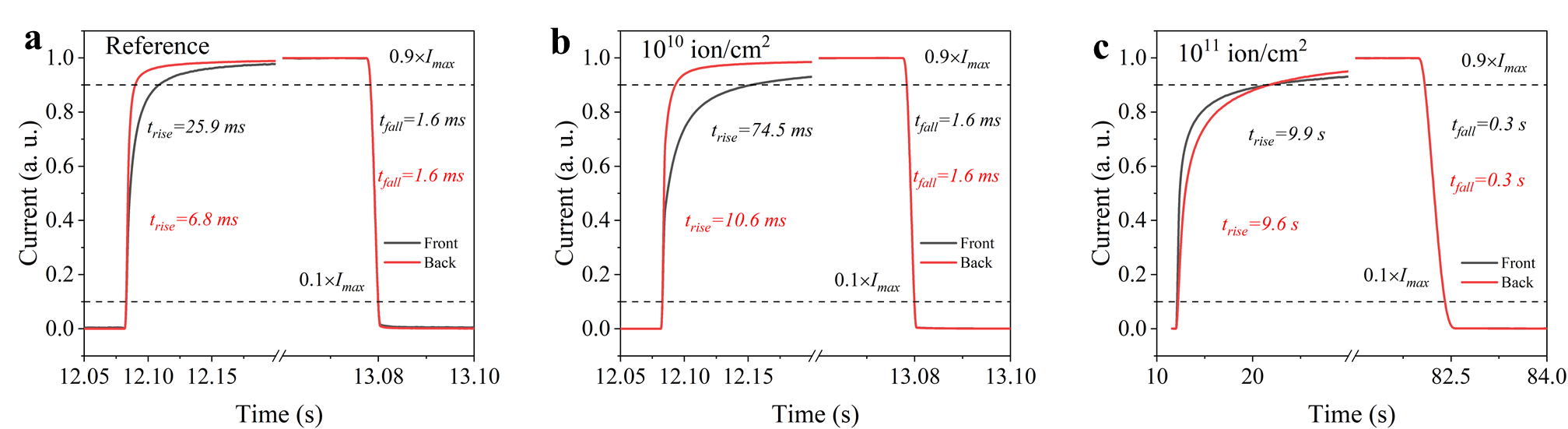


Figure S8. Temporal photoresponse of perovskite photodiodes with specific rise and fall times on both sides before (a) and after (b-c) irradiation.


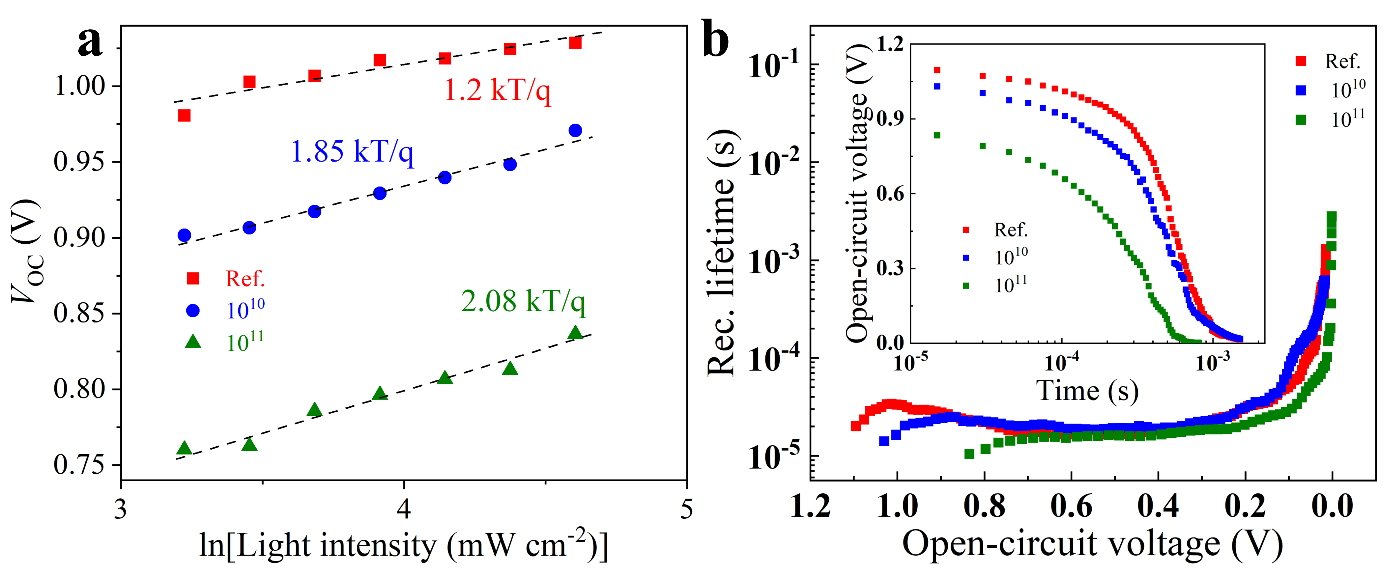


**Figure S9. (a) *V_oc_* vs. light intensity at different irradiation conditions; (b) recombination lifetime vs. *V_oc_* and dependence of *V_oc_* on measurement time (inset) for devices at different irradiation conditions.**


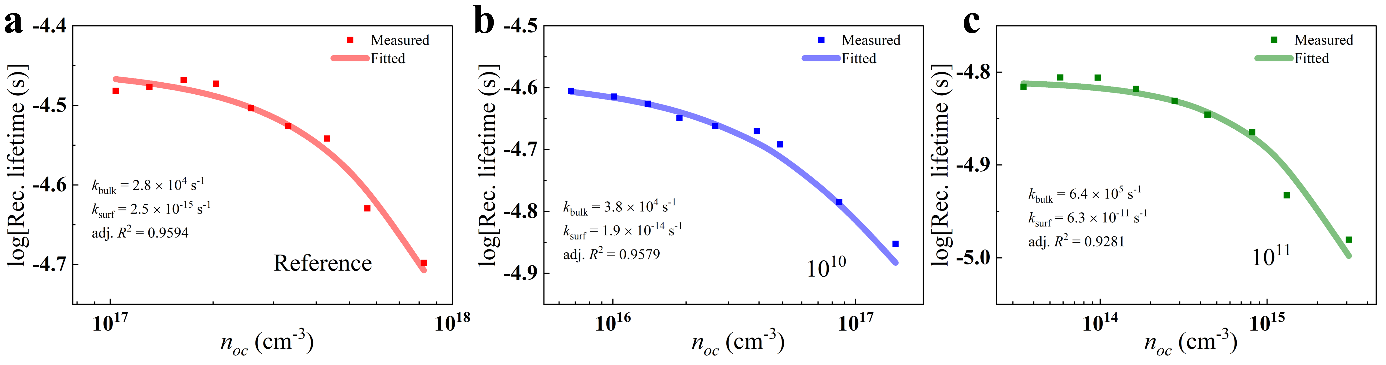


**Figure S10. Fitting of recombination lifetime vs. *n_oc_* for devices at different irradiation conditions.**

**Table S1.** Summarized characteristics of photodetectors under different conditions.

|  | *Illumination Side* | *R_max_*, A/W | *D^*^_max_*×10^11^, Jones | LDR, dB |
| --- | --- | --- | --- | --- |
| Reference | Front  Back | 0.39±0.01  0.33±0.01 | 2.4±0.16  2.0±0.48 | 63±1.14  56±2.16 |
| 10^10^ nucleons cm^-2^ | Front  Back | 0.37±0.01  0.31±0.01 | 4.4±0.19  3.7±0.48 | 60±2.28  56±2.2 |
| 10^11^ nucleons cm^-2^ | Front  Back | 0.25±0.02  0.18±0.03 | 2.8±0.69  2.2±0.56 | 57±3.7  48±3.87 |

**Table S2.** Comparison of the radiation stability of detectivity and responsivity, achieved in this work with different photodetectors based on compound semiconductors.

| Active layer | Type of Irradiation | Energy (MeV) | Fluence (particle cm^−2^) | Detectivity  *(D**_irrad_ */D**_0_) | Responsivity  *(R*_irrad_ */R*_0_) | Ref. |
| --- | --- | --- | --- | --- | --- | --- |
| Cs_0.05_MA_0.23_FA_0.72_Pb(I_0.77_Br_0.23_)_3_ | Xe ion | 231  231 | 10^10^  10^11^ | 1.78  1.19 | 0.95  0.65 | This work |
| Cs_0.04_Rb_0.04_(FA_0.65_MA_0.35_)_0.92_Pb(I_0.85_Br_0.14_Cl_0.01_)_3_ | Proton | 0.17  0.17 | 2·10^12^  10^13^ | 1.5  0.85 | 1.02  0.89 | ^[1]^ |
| CH_3_NH_3_PbI_3_ | Proton | 0.15  0.15 | 10^12^  10^13^ | 0.5  0.375 | 0.92  0.38 | ^[2]^ |
| β-Ga_2_O_3_/4H-SiC | Kr ions | 1302 | 1.6·10^10^ | 0.5 | 0.05 | ^[3]^ |
| Graphene oxide | Ag ions | 100 | 5·10^11^ | - | 0.67 | ^[3]^ |
| CdZnTe | Proton | 0.17 | 2·10^12^ | 0.17 | 0.45 | ^[4]^ |
| e-Ga_2_O_3_ | Proton | 0.15  0.15 | 5·10^12^  5·10^12^ | 0.26  0.11 | 0.62  0.32 | ^[5]^ |
| CNWs/CdZnTe | Proton | 1.5 | 10^12^ | 1.44 | 1.57 | ^[6]^ |
| a-Ga_2_O_3_/Si | Proton | 100 | 10^13^ | 0.37 | 0.24 | ^[7]^ |
| pc-Ga_2_O_3_/Si | Proton | 100 | 10^13^ | 0.4 | 0.16 | ^[7]^ |

Table S3. Parameters of ion irradiation.

| Ions | Energy, MeV/nucleon | Energy, MeV | Current (A) | Average Flux, cm^−2^ s^−1^ | Average Irradiation Time, s | Average Fluence, cm^−2^ |
| --- | --- | --- | --- | --- | --- | --- |
| ^132^Xe^23+^ | 1.75 | 231 | 3.05×10^-9^ | 5.75×10^7^ | 174 | 1.00×10^10^ |
| ^132^Xe^23+^ | 1.75 | 231 | 3.05×10^-9^ | 5.75×10^7^ | 1740 | 1.00×10^11^ |

References

[1] Solovan, M. M., Mostovyi, A. I., Aidarkhanov, D., Parkhomenko, H. P., Akhtanova, G., Schopp, N., Asare, E. A., Nauruzbayev, D., Kaikanov, M., Ng, A., & Brus, V. V., Extreme Radiation Resistance of Self-Powered High-Performance Cs0.04Rb0.04(FA0.65MA0.35)0.92Pb(I0.85Br0.14Cl0.01)3 Perovskite Photodiodes. **2023**, *Advanced Optical Materials*, *11*, 2203001. 10.1002/adom.202203001.

[2] Xiong, G., Qin, Z., Li, B., Wang, L., Zhang, X., Zheng, Z., Zhu, H., Zhao, S., Gao, J., Li, B., Yang, J., Li, X., Luo, J., Han, Z., Liu, X., & Zhao, F., Radiation Hardness and Abnormal Photoresponse Dynamics of the CH3NH3PbI3 Perovskite Photodetector. **2021**, *J. Mater. Chem. C*, *9*, 2095. 10.1039/D0TC05148A.

[3] Yang, Y., Zhang, Y., Ma, X., Li, Y., Zhang, F., Zhu, H., Li, D., & Wu, Z., Defect Engineering in β-Ga2O3/4H-SiC Heterojunctions via Kr Ion Irradiation for Enhanced Solar-Blind Photodetector Performance. **2025**, *ACS Appl. Mater. Interfaces*, *17*, 19996. 10.1021/acsami.5c01421.

[4] Solovan, M. M., Mostovyi, A. I., Parkhomenko, H. P., Kaikanov, M., Schopp, N., Asare, E. A., Kovaliuk, T., Veřtát, P., Ulyanytsky, K. S., Korbutyak, D. V., & Brus, V. V., A High-Detectivity, Fast-Response, and Radiation-Resistant TiN/CdZnTe Heterojunction Photodiode. **2023**, *Advanced Optical Materials*, *11*, 2202028. 10.1002/adom.202202028.

[5] Yang, Y., Zhu, H., Wang, L., Jiang, Y., Wang, T., Liu, C., Li, B., Tang, W., Wu, Z., Yang, Z., & Li, D., In-Depth Investigation of Low-Energy Proton Irradiation Effect on the Structural and Photoresponse Properties of ε-Ga2O3 Thin Films. **2022**, *Materials & Design*, *221*, 110944. 10.1016/j.matdes.2022.110944.

[6] Yerlanuly, Y., Parkhomenko, H. P., Zhumadilov, R. Y., Nemkayeva, R. R., Akhtanova, G., Solovan, M. M., Mostovyi, A. I., Orazbayev, S. A., Utegenov, A. U., Ramazanov, T. S., Gabdullin, M. T., Jumabekov, A. N., & Brus, V. V., Achieving Stable Photodiode Characteristics under Ionizing Radiation with a Self-Adaptive Nanostructured Heterojunction CNWs/CdZnTe. **2023**, *Carbon*, *215*, 118488. 10.1016/j.carbon.2023.118488.

[7] Kaur, D., Vashishtha, P., Khan, S. A., Kulriya, P. K., Gupta, G., & Kumar, M., Phase Dependent Radiation Hardness and Performance Analysis of Amorphous and Polycrystalline Ga _2_ O _3_ Solar-Blind Photodetector against Swift Heavy Ion Irradiation. **2020**, *Journal of Applied Physics*, *128*, 065902. 10.1063/5.0019786.
